# Intra-Arterial Chemotherapy (Ophthalmic Artery Chemosurgery) for Group D Retinoblastoma

**DOI:** 10.1371/journal.pone.0146582

**Published:** 2016-01-12

**Authors:** David H. Abramson, Anthony B. Daniels, Brian P. Marr, Jasmine H. Francis, Scott E. Brodie, Ira J. Dunkel, Y. Pierre Gobin

**Affiliations:** 1 Ophthalmic Oncology Service, Memorial Sloan Kettering Cancer Center, New York, New York, United States; 2 Department of Ophthalmology and Visual Sciences, Vanderbilt University Medical Center, Nashville, Tennessee, United States; 3 Department of Cancer Biology, Vanderbilt University, Nashville, Tennessee, United States; 4 Department of Radiation Oncology, Vanderbilt University Medical Center, Nashville, Tennessee, United States; 5 Vanderbilt-Ingram Cancer Center, Vanderbilt University Medical Center, Nashville, Tennessee, United States; 6 Department of Ophthalmology, Mount Sinai School of Medicine, New York, New York, United States; 7 Department of Pediatrics, Memorial Sloan Kettering Cancer Center, New York, New York, United States; 8 Department of Pediatrics, Weill Cornell Medical College, New York, New York, United States; 9 Neurosurgery / Interventional Radiology, Weill Cornell Medical College, New York, New York, United States; Massachusetts Eye & Ear Infirmary, Harvard Medical School, UNITED STATES

## Abstract

**Purpose:**

To report globe salvage rates, patient survival and adverse events of ophthalmic artery chemosurgery (OAC) for International Classification of Retinoblastoma (ICRB) group D retinoblastoma (naive and after prior failures).

**Methods:**

Single institution retrospective review of all Group D eyes treated with OAC from 5/2006-12/2012. Patients were treated according to our previously-published techniques. Primary outcome was globe retention without need for external beam radiotherapy (EBRT). Demographics, prior treatments, OAC agents used, and adverse events were also recorded.

**Results:**

112 group D eyes (103 patients) that underwent OAC were included (average follow-up was 34 months, range: 2–110 months). 47 eyes were treatment-naïve, 58 eyes received prior treatments elsewhere, and 7 young infants (7 eyes) underwent our published “bridge therapy” (single agent intravenous carboplatin) until old enough to undergo OAC. Median number of OAC sessions/eye was 3 (range 1–9). 110/112 eyes received intra-arterial melphalan, but only 31 eyes received melphalan alone. 43 eyes received carboplatin, and 78 eyes received topotecan (never as a single agent). 80/112 eyes received >1 drug over their treatment course, and 39 eyes received all three agents. 24 eyes (16 pretreated, 7 treatment-naïve, 1 bridge) failed treatment and required enucleation during the study period. Enucleation and EBRT were avoided in 88/112 eyes (78.6%; including 40/47 [85.1%] treatment-naïve eyes, 42/58 [72.4%] previously-treated eyes, and 6/7 eyes [85.7%] among bridge patients). By Kaplan-Meier survival analysis, globe salvage rate was 74% at 110 months among all patients, and 85% at 110 months in the treatment-naïve subgroup. Transient grade 3/4 neutropenia was more common in patients receiving OAC bilaterally. No child died of metastatic disease.

**Conclusions:**

OAC is effective for curing group D retinoblastoma, achieving rates of globe salvage many times higher than systemic chemotherapy (10–47%), even in eyes that previously failed other treatments. OAC can be performed multiple times, using multiple agents, on one or both eyes of patients.

## Introduction

Over the past two decades, the introduction of systemic chemotherapy for the management of intraocular retinoblastoma has enabled globe retention for these children without the need for external beam radiation or enucleation.[1–3] Using primary systemic chemotherapy, most of the successes have been in children with International Classification of Retinoblastoma (ICRB) groups A, B and C eyes.[4] Success rates of systemic chemotherapy for group D eyes remain poor, with published rates of ocular survival of only 10–47%,[4–13] and group E eyes are nearly always enucleated.

We first began to perform intra-ophthalmic artery chemotherapy (ophthalmic artery chemosurgery, OAC) at Memorial Sloan Kettering Cancer Center (MSKCC) in 2006.[14] Since that time, we have performed the procedure over 1500 times, and OAC is now being performed in more than 45 countries worldwide. OAC allows many eyes, which previously would have required enucleation, to be saved, including many group E eyes.[15]

Since the previous standard of care, systemic chemotherapy, had relatively poor success at saving group D eyes, we wanted to assess our success rates in treating these eyes using our OAC technique. In addition, intravenous chemotherapy is associated with many systemic side effects, including neutropenia during treatment and possibly secondary acute myelogenous leukemia (sAML) years later.[1, 5, 11, 16–18] However, complications related to OAC have likewise been reported by various authors.[19, 20] Here we report our success rates with OAC both as primary treatment, as well as for patients who had previously failed other forms of treatment, for group D retinoblastoma. We demonstrate rates of globe salvage with OAC that are much higher than those reported previously using other previous treatment modalities.

## Material and Methods

### Patients

This was a retrospective review of all patients treated at MSKCC from May 2006 until December 31, 2012. Inclusion criteria were patients with ICRB group D retinoblastoma in at least one eye at the time of presentation to MSKCC, who received OAC for the management of the group D eye. For all patients, records included the initial presentation and ICRB and Reese-Ellsworth classification, the presence or absence of subretinal or vitreous seeds, whether the patient had unilateral or bilateral disease or a positive family history of RB, the contralateral eye’s classification (for bilateral cases), the presenting ERG amplitude, whether or not any previous treatments had been given elsewhere, and the details of any prior treatments (see S1 Table). Ocular and patient outcomes and most recent ERG amplitude were also recorded. For patients prior to 2010 who were initially classified under the Reese-Ellsworth classification system, RetCam fundus photos and large-scale drawings in the patients charts were reviewed in assigning each patient an ICRB group. The Children’s Oncology Group (COG) version of the ICRB was used in all cases. Patients who were not treated with OAC due to patient/family refusal of this technique were not included.

### OAC Technique

OAC was performed according to our published techniques.[14, 15, 21–25] Briefly, under general anesthesia and with the patient heparinized, a catheter was inserted into the femoral artery and advanced into the internal carotid and up to (but not in to) the os of the ophthalmic artery. Catheter tip position was confirmed by fluoroscopy, and care was taken to avoid placing the tip directly into the ophthalmic artery, to avoid wedge flow. Once position was confirmed, (each) drug was injected in a pulsatile fashion over ten minutes, as published previously.[26–28] For patients receiving bilateral sequential OAC (tandem therapy),[29] the catheter was then retracted and advanced to the os of the contralateral ophthalmic artery, position reconfirmed and drug re-injected. Cerebral angiography was performed at the end of the procedure to ensure no vascular flow anomalies were present in the brain. Intranasal epinephrine was used to reduce collateral blood flow to the nasal mucosa, and topical neosynephrine drops were used to minimize forehead hyperemia, rash and edema in the distribution of the supratrochlear artery.[30] Patients receiving bilateral treatments[29] or triple therapy with melphalan, carboplatin and topotecan[28] usually received IV steroids at the time of treatment, and an oral steroid taper thereafter. For patients with anomalous vascular anatomy, alternate techniques to infuse into the ophthalmic artery were used, according to our previously published techniques.[24] General starting doses were: melphalan 0.4 mg/kg (up to a starting dose of 5 mg), carboplatin 50 mg (which could be increased subsequently if the child’s vascular anatomy demonstrated an abnormal amount of collateral flow to extraocular structures), and topotecan 0.2–4 mg (patients treated earlier in the study tended to receive lower doses). These doses could be increased or decreased for subsequent infusions for each patient based on the judgment of the treating ocular oncologist and in real time by the interventional radiologist. The choice of drugs was decided by the treating ocular oncologist, but as a general rule, more severe tumors received a combination of multiple agents. For bilateral treatments, the worse eye usually received melphalan initially, and the contralateral eye received carboplatin. Parents were instructed to have a complete blood count with differential performed 7–10 days after the procedure. Patients were examined under anesthesia three to four weeks later, and treatments were performed as frequently as every three to four weeks, when necessary. Focal consolidation with transpupillary thermotherapy or cryotherapy was performed at the time of examination under anesthesia.

Details of all procedures were recorded, including catheter type, any vascular flow anomalies, drug regimens and dosing, any adverse events, and blood counts. Final outcome was recorded at most recent follow-up, up to the predetermined study end date (December 31, 2012). Success was defined as globe retention, without the need for external beam radiotherapy (EBRT), at the study end date.

The Memorial Sloan Kettering Cancer Center Institutional Review Board approved this study. This study was performed in accordance with the Declaration of Helsinki, and with the Health Insurance Portability and accountability Act (HIPAA). All patients provided written consent for all procedures described herein.

### Statistical Analyses

Statistical analyses were performed using the R (Version 3.0.3) statistical software, with the assistance of biostatisticians Drs. Yu Shyr, Fei Yu and Liping Du of the Vanderbilt Center for Quantitative Sciences of the Vanderbilt Department of Biostatistics. Kaplan-Meier survival analysis curves were created using STATA.

## Results

### Characteristics of Patients and Eyes Enrolled

Our retrospective review included 112 group D eyes of 103 consecutive patients who received OAC for management of the group D eye. Follow-up was for an average of 34 months (range: 2–110 months). 53 patients (51%) were female and 50 (49%) were male. 63 patients (61%) had bilateral disease and 40 patients (39%) had unilateral disease. Of the 112 group D eyes, 56 (50%) were right eyes and 56 (50%) were left eyes. By Reese-Ellsworth classification, 85/112 (76%) eyes were group Vb, 17/112 (15%) were group Va, 3 (3%) were group IV, 4 (4%) were group III, 3 (3%) were group II, and none were group I. 85/112 (76%) of eyes had vitreous seeds. Among eyes in patients with bilateral disease, the contralateral eye’s ICRB classification could be determined in 58/72 (81%) of eyes. In 14 patients/eyes, the contralateral eye could not be classified due to prior enucleation of the other eye elsewhere, without adequate information to assign a classification. Of the 58 eyes for whom the contralateral eye could be classified, the contralateral eye was ICRB group A for 4 eyes, group B in 8 eyes, C in 15 eyes, D in 20 eyes and E in 11 eyes.

47/112 eyes were treatment-naïve at the time they received OAC with us, 58 eyes had received prior treatment and 7 eyes received our previously-described “bridge” chemotherapy protocol.[31] The bridge approach was used in patients, usually under the age of 3 months. These infants received single agent intravenous (IV) carboplatin until they grew large enough to treat with OAC. The average age in the treatment-naïve group was 18.9 months (median age 10 months, range 3–111 months), and the average age in the pretreated group was 37.3 months (median age 24 months, range 5–252 months; p<0.001, Wilcoxon rank sum).

### Previous Treatment Regimens

**Fig 1:**
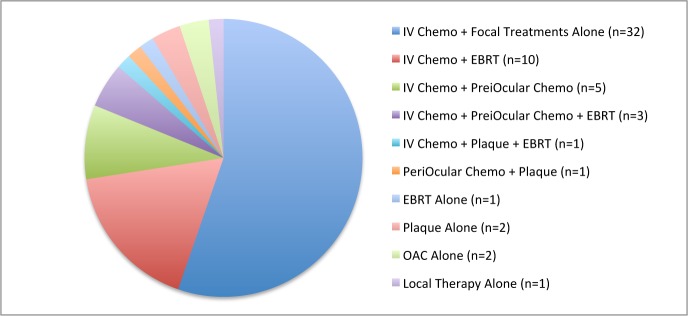
Previous treatment strategies, by eye.

Among the 58 pretreated (non-bridge) eyes, 51/58 eyes (88%) had previously received IV chemotherapy, 15/58 (26%) had received prior EBRT, 9 eyes (16%) received periocular chemotherapy, 4 (7%) received plaque brachytherapy and 1 eye (2%) had only been treated previously with cryotherapy. 2 eyes (3%) had received and failed OAC at a different institution prior to being treated at MSKCC. Many patients had received combinations of treatments. Overall, the previous treatment strategies for the 58 pretreated patients were variable (most patients also received focal therapy with laser or cryotherapy in conjunction with the following). The most common treatment strategy was systemic chemotherapy (plus focal therapies) alone, utilized in 32/58 eyes (55%), usually with the standard vincristine/etoposide/carboplatin (VEC) regimen. The distribution of treatment strategies is shown in Fig 1.

### OAC Treatments Received

**Fig 2:**
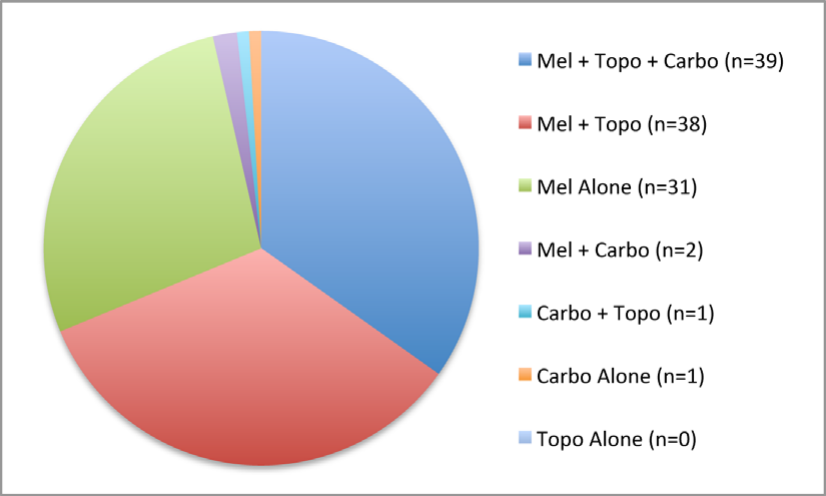
Distribution of eyes receiving different combinations of OAC chemotherapy agents.

On average, eyes received 3.7 OAC treatments (median 3, range 1–9). Treatment-naïve eyes received an average of 3.5 treatments (median 3, range 1–9), pretreated eyes received an average of 3.9 treatments (median 3, range 1–9), and eyes of patients that received bridge chemotherapy received an average of 3.1 OAC treatments (median 3, range 1–5; p = 0.25, Wilcoxon rank sum). Accounting for the fact that eyes could receive combinations of chemotherapy per infusion, treatment-naïve eyes received an average of 6.1 drug infusions (median 6, range 1–20), pretreated eyes received an average of 7.1 drug infusions (median 6, range 1–21), and eyes of patients that received bridge chemotherapy received an average of 4.6 OAC drug infusions (median 4, range 1–9; p = 0.29, Wilcoxon rank sum). 110/112 eyes (98%) received melphalan as a part of their OAC treatment at some point, but only 31/112 eyes (28%) received melphalan as the only agent used. 78 eyes (70%) received topotecan, but never as a single agent. 43 eyes received carboplatin, but only 1 eye received carboplatin alone. Over the course of their OAC treatments, 39 eyes (35%) received all three agents. Other combinations were used, and the distribution of OAC drug combination regimens is shown in Fig 2. No eye received intravitreous chemotherapy.

### Success Rates with OAC

**Fig 3:**
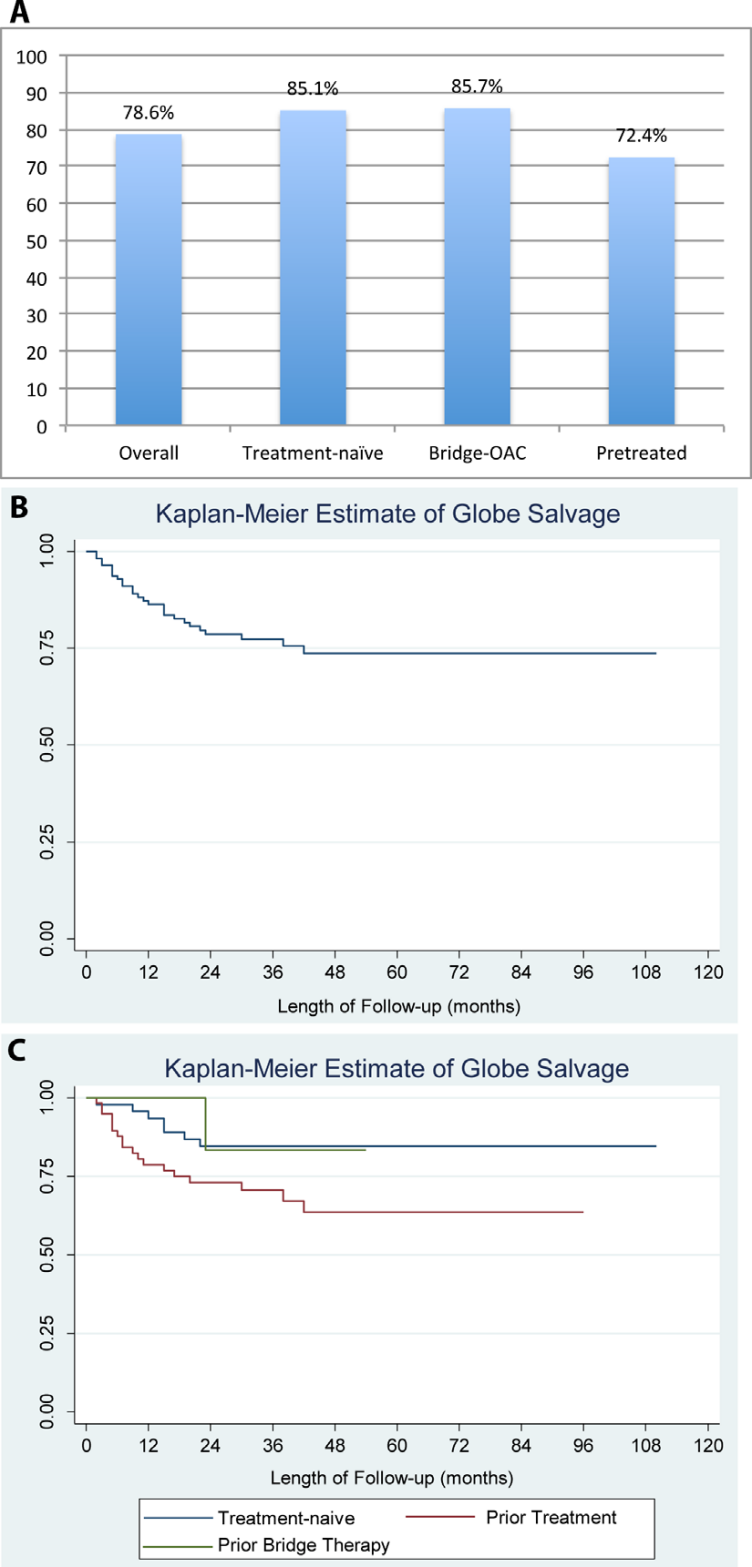
OAC treatment success rates. Success was defined as globe salvage without the need for EBRT. **A)** OAC treatment success rates by treatment group. **B)** Kaplan-Meier survival analysis for all group D eyes receiving OAC, and **C)** by subgroup (prior treatment, treatment-naïve, and “bridge” chemotherapy).

Overall, enucleation and EBRT were both avoided in 88/112 (78.6%) of all group D eyes treated with OAC. Among treatment-naïve eyes, the success rate was 40/47 eyes (85.1%), among bridge-OAC patients, the success rate was 6/7 eyes (85.7%), and among eyes that had previously failed other treatments elsewhere, the success rate was 42/58 eyes (72.4%; Fig 3A). There was a trend toward a higher success rate in the OAC and bridge-OAC groups compared to the pretreated group (p = 0.1, Pearson) but this did not reach statistical significance. Kaplan-Meier survival analysis showed an overall globe salvage rate of 74% at 110 months (Fig 3B). For the treatment-naïve subgroup, this globe salvage rate was 85% at 110 months (Fig 3C).

Essentially all patients (although not all tumors) who received OAC had focal consolidation with laser or cryotherapy. 33 eyes had some other form of treatment in addition to the OAC with focal consolidation. 3 eyes received periocular chemotherapy. 12 eyes had radioactive plaque brachytherapy (1 was in an eye that also received periocular chemotherapy). 2 eyes received systemic chemotherapy for treatment of the group D eye, and one of these eyes (which also received radioactive plaque brachytherapy) was able to avoid enucleation, 2 eyes (one pretreated and one treatment-naïve) received EBRT, but both ultimately required enucleation, as EBRT could not salvage either of the eyes that failed OAC. Overall, 24 eyes (7 treatment naïve, 1 bridge eye, and 16 eyes that had previously failed other treatments before receiving OAC) were ultimately enucleated. Of the 33 eyes that received additional treatment in conjunction with OAC, 10 were saved. Of the 10 eyes that received additional treatment and ultimately did not require enucleation, 9/10 received treatment for a focal lesion with plaque brachytherapy.

### Adverse Events

Out of 103 patients treated with OAC, 39 patients developed grade 3/4 neutropenia. Of the 39 patients who developed grade 3/4 neutropenia, 27 were patients with bilateral retinoblastoma, and 17 received OAC for both eyes. Of the 76 patients that either had unilateral disease, or who only received OAC in one eye, only 22 developed grade 3/4 neutropenia at some point during their treatment course. In contrast, of the 27 patients who received bilateral OAC, 17 developed grade 3/4 neutropenia at some point during their treatment (OR = 4.2, p = 0.0026, Fisher exact). There was no significant association of grade 3 or 4 neutropenia with total number of treatments for the group D eye, with the total number of drug infusions the eye received, or with the number of triple agent (melphalan, carboplatin, plus topotecan) infusions that the eye received.

In addition, out of 103 patients treated with OAC, 44 experienced (usually mild) bronchospasm at some point during the actual OAC treatment. There was allergy-type reaction in 5 patients, grade 3 or 4 thrombocytopenia in 4, fever in 4, cardiorespiratory side effects in 3, injection site complications (such as thrombosis or bleeding) in 3, and epistaxis in 1 patient.

Of 112 eyes treated with OAC, 44 (39%) experienced a local/regional adverse event at some point during their treatment course. The most common adverse event was eyelid edema or localized skin erythema (25), which was usually transient and self-limited. Other adverse events included madarosis (10), retinal or choroidal vascular occlusions (6), phthisis (5), vitreous hemorrhage (4), ptosis (4), optic nerve swelling (3), retinopathy (2), cranial nerve palsy (2), ophthalmic artery vessel injury or sclerosis (2), and suprachoroidal hemorrhage (1).

Three patients, who were all in the treatment-naïve group, developed metastases over the course of their follow-up, but all three were successfully treated for their metastatic disease and all survived. One child developed (and died from) a second, non-ocular cancer (pineal gland). No patient developed a new intraocular tumor during treatment or follow-up, as we have previously reported for OAC.[32]

## Discussion

In our study, we demonstrated high rates of successful globe salvage in eyes treated with OAC (without supplemental intravitreous chemotherapy), with an overall success rate of 78.6% (88/112 eyes saved). This was true across groups: in eyes that received OAC as primary treatment (40/47 eyes, 85.1%), in eyes that received carboplatin bridge to OAC because the infant was too young to receive OAC directly at the outset (6/7 eyes, 85.7%) as well as in eyes that had previously failed other treatments elsewhere (42/58 eyes, 72.4%).

Our data also demonstrates that high success rates can be achieved with OAC even in eyes that have previously failed other treatment modalities. It is interesting that the success rate for the pretreated group is numerically (but not statistically significantly) lower than for the treatment-naïve group. There are at least two possible reasons why this might be so. The first hypothesis is that the eyes inherently do better if OAC is initiated from the outset.[21] By this argument, the time spent receiving other treatments in an attempt to cure the eye allows the tumor to progress to a stage that is inherently more resistant to treatment with subsequent OAC. This might happen, for example, by accumulating more mutations that allow for clonal escape from systemic chemotherapy, but also allows for cross-resistance to subsequent OAC that might otherwise have been curative. The alternative explanation is that these eyes, which failed prior alternative treatment strategies, therefore represent those eyes that are most difficult to cure from the outset. By this argument, the easiest-to-cure eyes were already cured by systemic chemotherapy (or another treatment modality), and therefore never entered into this study to receive OAC in the first place. Stated another way, our pretreated group is biased toward those eyes that are inherently more resistant to treatment. The success rate of 72.4% in the pretreated group therefore demonstrates the power of OAC to achieve cure even in these difficult to treat eyes that have failed other regimens previously.

We also found that our previously-described “bridge” strategy,[31] whereby patients who are too young and have femoral arteries too small to be safely accessed are treated with single agent systemic carboplatin as a bridge to planned OAC, do just as well as those who are old and large enough to receive OAC from the outset. Therefore, treating clinicians should not choose alterative chemotherapy approaches in young infants simply for fear that the couple month delay (usually until age 3 months) until OAC is possible in these infants might somehow lead to deleterious outcomes. It should be noted however, that in our bridge group the intention from the outset was to transition all patients to OAC as soon as it was technically feasible to do so. Therefore, patients should not be delayed and receive continued single agent carboplatin with intention to ultimately cure the child with intravenous monotherapy, rather, it should only be given until OAC is possible beginning at three months of age.

**Fig 4:**
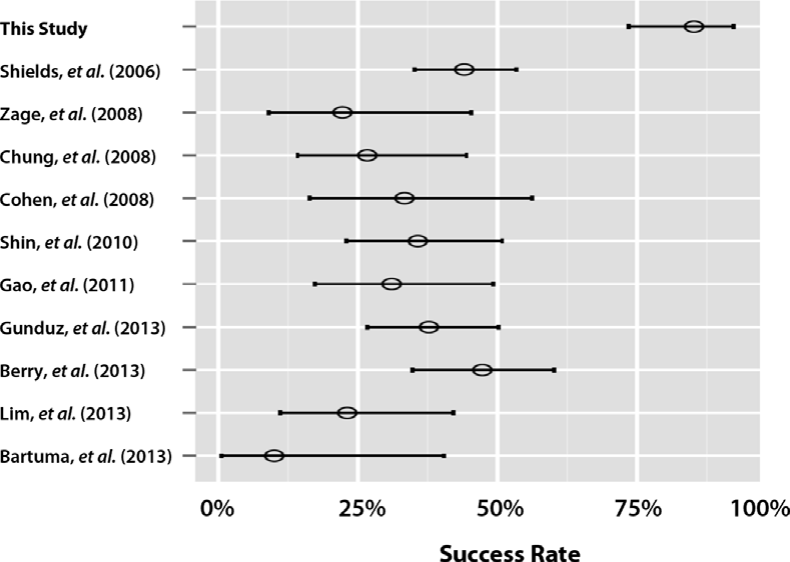
Comparison of our OAC success rates to previously-published success rates using systemic chemotherapy with or without focal consolidation. Success was defined as globe salvage without the need for EBRT. (Percent success +/- 95% confidence intervals).

There is scant literature on treatment of Group D eyes after failed treatment because they are routinely enucleated.[2, 3, 11, 13] The best comparator to systemic chemotherapy success rates published in the literature is with our treatment-naïve group. The above rates of success are significantly higher than the success rates published in the literature for globe salvage with systemic chemotherapy in conjunction with focal consolidation (Fig 4). The published rates of globe salvage (without the need for EBRT) in the literature range from 10–47% (weighted average = 36.7% success) depending on the series and treating center (Table 1).[4–13] In comparison, our treatment-naïve group’s success rate was significantly higher (85.1%; Fig 4).

**Table 1:** Comparison to published success rates of chemotherapy with or without focal consolidation or other local treatments.

Authors	Year	Treatment	Total # Group D Eyes	Eyes Successfully Treated	Success Rate
Shields *et al*.	2006	Chemo/Focal	109	48	47%
Zage *et al*.	2008	Chemo/Focal	18	4	22%
Chung *et al*.	2008	Chemo/Focal	30	8	27%
Cohen *et al*.	2008	Chemo/Focal	18	6	33%
		+/- Plaque			
Shin *et al*.	2010	Chemo +/- Focal	42	15	36%
Gao *et al*.	2011	Chemo/Focal	29	9	31%
Gunduz *et al*.	2013	Chemo/Focal	61	23	38%
		+/- Plaque			
Berry *et al*.	2013	Chemo/Focal/ Periocular Chemo	55	26	47%
Lim *et al*.	2013	Chemo/Focal	26	6	23%
Bartuma *et al*.	2013	Chemo/Focal	10	1	10%
		+/- Plaque			

We decided on a strict cut off study follow-up end date of December 31, 2012, for all patients. Therefore, all primary end point (globe salvage) determinations are based on the last available follow-up information or the globe status on the above date, whichever is earlier. The reason for this study end date is that, beginning in early 2013, we began to use intravitreal melphalan widely, in conjunction with OAC, for the treatment of persistent vitreous seeds.[33–35] Most eyes that fail OAC treatment do so because of persistent vitreous seeds, and intravitreal melphalan appears to be particularly effective at treating vitreous seeds. Therefore, had we included follow-up into 2013, it would be hard to ascertain whether the success was from OAC or from the addition of intravitreal chemotherapy.

Eyes were treated with the minimum number of cycles of OAC necessary either to induce total regression of the tumor, or to shrink the tumor sufficiently that local consolidation with transpupillary thermotherapy or cryotherapy could be applied. Once no active tumor was noted on exam, OAC treatments were stopped. The large range of treatment sessions (3–9) derived from the variability in group D eyes. Eyes classified as Group D based on the presence of vitreous seeds tended to require more intensive treatment (either higher doses or multiple agents), as well as a greater number of treatments, than eyes that were classified as Group D based on extensive subretinal seeding, as subretinal seeds are quite easily treated with OAC. Similarly, more vitreous seeds tended to require more treatment sessions compared to eyes with fewer vitreous seeds, as this cohort was prior to the introduction of intravitreal chemotherapy at our institution.

We considered OAC treatment failure to be any eye that required enucleation or required EBRT. We considered EBRT to be treatment failure because EBRT has been shown to increase the risk of second cancers in patients with germline RB.[36, 37] Plaque brachytherapy, due to its localized radiation field, has not been associated with the subsequent development of second cancers, and so the addition of plaque brachytherapy was not considered to denote a failure of OAC, but rather the application of a focal treatment.[38] While several patients had received, and failed, EBRT prior to being referred to us for OAC, in our series, only two patients received post-OAC EBRT as salvage therapy, both of whom were treated with OAC early in this series, and both of whom received EBRT upon returning to their original referring institution. Interestingly, EBRT was not able to salvage the globe successfully in either of these two patients, and both required subsequent enucleation. In the past, there has been concern expressed about the radiation exposure due to the fluoroscopy required for OAC. We have previously published our various techniques that allow us to minimize radiation dose during OAC treatments.[39]

Out of 112 group D eyes treated with OAC, 24 eyes were not salvaged. This included 7 treatment-naïve eyes, 16 eyes that had received prior treatment elsewhere, and 1 eye treated with bridge chemotherapy. Of the 16 previously treated eyes that ultimately required enucleation, 14 had been treated with IV chemotherapy previously, 6 had received EBRT prior to referral to us (5 of which had received both IV chemotherapy and EBRT). Of these 16 eyes, 1 received neither IV chemotherapy nor EBRT, but instead had been treated with plaque brachytherapy in combination with laser and cryotherapy.

In a multivariate analysis, we were not able to identify specific predictors of failure with OAC. This might be due to the overall high success rate with OAC. Put another way, there simply were not enough eyes that failed OAC to be able to tease out the factors that predicted that failure. Similarly, we could not find any statistically significant associations between prior treatment strategies and subsequent OAC failure, in part because of the low rate of failure even in the pretreated group and because almost all pretreated eyes received intravenous chemotherapy. This also raises the question of whether IV chemotherapy should ever be used as primary treatment,[40] or whether OAC as primary treatment might be a better strategy.[21]

The published literature on systemic chemotherapy for the treatment of RB often does not include a discussion of adverse events, either in the eye or for the patient systemically.[41] The main side effects associated with the systemic chemotherapy agents most commonly used are neutropenia and neutropenic fever,[1, 6, 11, 13] other forms of myelosuppression,[42, 43] thrombocytopenia,[43] ototoxicity,[44, 45] nephrotoxicity,[8] neurotoxicity, potential reduction in fertility, and the subsequent development of secondary acute myelogenous leukemia (sAML).[1, 5, 11, 16–18] Ramasubramanian *et al*. found no statistically significant reduction (p = 0.1) in the incidence of pineoblastoma between germline retinoblastoma patients treated with systemic chemotherapy and those not treated with systemic chemotherapy. In our study, 1/47 patients with bilateral retinoblastoma developed a pineoblastoma. This is not statistically different from the rate found by Ramasubramanian *et al*. in their systemic chemoreduction group (1/252 patients, p = 0.29, Fisher’s exact test).

Murphree *et al*. have written that “transient myelosuppression occurs in virtually all cases [of retinoblastoma treated with systemic chemotherapy].”[42] Febrile neutropenia requiring hospitalization has been variably reported following systemic chemotherapy for RB in 12.7%,[11] 22.5%,[1] 28.6%,[6] 38%[13] and 80%[13] of patients, depending on the series and on the specific chemotherapeutic regimen utilized. In contrast, only 4/103 patients (3.9%) who received OAC in this study developed a fever. Reporting of reduction in fertility in these published studies is hampered by the fact that this would only become apparent decades later. Since systemic chemotherapy only became widely used in the early- to mid-1990s, these patients would only be beginning to try to establish families now. Therefore, the true rate of infertility associated with the use of systemic chemotherapy for RB has not yet become manifest. Similarly, while the development of secondary hematologic malignancies has been reported by multiple authors[1, 5, 11, 16–18] following systemic chemotherapy for RB, the total number of children who will develop sAML in the future remains to be seen. While nephrotoxicity has not been commonly reported, there have been reported deaths associated with chemotherapy-induced nephrotoxicity causing Fanconi syndrome.[8]

We did find that patients who received bilateral (tandem or sequential) OAC for the treatment of bilateral RB were more likely to develop grade 3 or 4 neutropenia at some point during their treatment course but few required transfusion. Patients with unilateral RB or bilateral RB patients who received OAC for only one eye were much less likely to develop neutropenia. Interestingly, there was no significant association between the development of neutropenia and the total number of OAC treatments the eye received, nor with the total number of drug infusions the eye received, nor with the number of triple agent (melphalan, carboplatin, plus topotecan) infusions that the eye received. Fever associated with neutropenia, or myelosuppression or cytopenias requiring hospitalization, were extremely rare following OAC in our study.

## Conclusions

In conclusion, OAC is a very effective treatment for curing ICRB group D retinoblastoma, and appears to achieve rates of globe salvage many times higher than systemic chemotherapy (and, of course, higher than primary enucleation), without compromising patient survival. The procedure can be performed safely multiple times, using multiple intra-arterial chemotherapeutic agents, on one or both eyes of patients, and allows the vast majority of children to keep their eyes. It is well tolerated by patients with an acceptable side effect profile, consisting mostly of local side effects that are mild and transient (although rare sight threatening consequences do occur). High success rates can even be achieved in eyes that previously failed other treatment modalities, but our data suggest that beginning treatment primarily with OAC, rather than only using it as salvage therapy after failed systemic chemotherapy, might possibly be a better strategy. However, this is a single center, retrospective study, and a prospective randomized study would be required to answer this question definitively.

## Supporting Information

S1 Table Complete de-identified data set used for analyses. (PDF)
