# Supplementary material for: Intra-Arterial Chemotherapy (Ophthalmic Artery Chemosurgery) for Group D Retinoblastoma
Source: PLoS One. 2016 Jan 12;11(1):e0146582. doi: 10.1371/journal.pone.0146582 (PMC4710506; doi:10.1371/journal.pone.0146582)
Supplement: S1 Table — (PDF) [file pone.0146582.s001.pdf]

Patient ID	Gender	Age at tx (months)	Unilat Bilat*	Vitreous Seeds (1=Y, 0=N)	Reese-Ellsworth	StudyEye (OD=0, OS=1)	Other Eye Received IAC? (0=Unilateral, 1=No, 2=Yes)	Prior Rx (0=naïve, 1=yes, 2=bridge)	Prior Systemic Chemotherapy (0=N, 1=Y, 2=Bridge)	Number of Previous IV Chemo Cycles	Prior Periocular Chemo (1=Y, 0=N)	Prior EBR (1=Y, 0=N)	Prior Plaque (1=Y, 0=N)	Prior Laser Therapy (1=Y, 0=N)	Prior Cryo Therapy (1=Y, 0=N)	Prior IAC Elsewhere	# IA Prcedures/pt	# IA Procedures/eye	IAC Drugs Used	AE Bronchospasm	AE Eyelid edema or rash	AE Lash loss	AE Retinal or Choroidal Vascular	AE Retinopathy	AE G3/4 Neutropenia	AE Phthisis	AE CN Palsy	AE Direct vessel injury	AE Allergy/Immune	AE Vit Heme	AE Suprachoroidal hemorrhage	AE Optic Nerve	AE Ptosis	Additional Treatment After (Exc Focal)	Periocular Chemo (1=Y, 0=N)	Plaque (1=Y, 0=N)	IV Chemo (1=Y, 0=N)	EBR (1=Y, 0=N)	Enucleation (1=Y, 0=N)	Primary endpoint (req enuc or EBR) (0=No, 1=Yes)	Mets	Death	New Ocular Tumors	Second Nonocular Cancers	Follow-up (months)
1	M	18	B	1	5B	1	1	1	1	6	0	0	0	1	1	0	3	3	M,T	1	0	0	0	0	0	0	0	0	1	0	0	0	0	0	0	0	0	0	0	0	0	0	0	0	8
2	F	10	B	1	5B	1	1	1	1	6	0	0	0	1	1	0	3	3	M	0	0	0	0	0	0	0	0	0	0	0	0	0	0	0	0	0	0	0	0	0	0	0	0	0	26
3	F	10	B	0	4A	1	2	0	0	0	0	0	0	0	0	0	2	2	M,T	0	0	0	0	0	0	0	0	0	0	0	0	0	0	0	0	0	0	0	0	0	0	0	0	0	22
4	F	15	B	1	5B	1	2	1	1	6	0	0	0	0	0	0	8	8	M,T,C	1	0	0	0	0	1	0	1	1	0	0	0	0	1	1	0	0	0	0	1	1	0	0	0	0	11
5	M	31	B	1	5B	0	1	1	1	7	0	1	0	0	0	0	6	6	M,T,C	0	0	0	0	0	0	0	0	0	0	0	0	0	0	1	0	0	0	0	1	1	0	0	0	0	10
6	M	3	B	1	5B	0	1	2	2	2	0	0	0	1	0	0	5	5	M,T,C	1	0	1	0	0	1	0	0	0	0	0	0	0	0	0	0	0	0	0	0	0	0	0	0	0	54
7	F	3	U	0	5B	1	0	0	0	0	0	0	0	0	0	0	3	3	M	1	0	0	0	0	1	0	0	0	0	0	0	0	0	0	0	0	0	0	0	0	0	0	0	0	48
8	F	34	B	1	5B	0	1	1	1	6	0	1	0	1	1	0	3	3	M	0	1	0	1	0	0	1	0	0	0	0	0	0	0	0	0	0	0	0	0	0	0	0	0	0	88
9	F	7	B	1	5B	1	2	0	0	0	0	0	0	0	0	0	4	4	M,T,C	1	0	0	0	0	1	0	0	0	0	0	0	0	0	0	0	0	0	0	0	0	0	0	0	0	39
10	F	33	B	1	5B	0	2	1	1	4	0	1	0	1	1	0	7	4	M,T	1	1	1	0	0	1	0	0	0	0	0	0	0	0	0	0	0	0	0	0	0	0	0	0	0	47
11	F	8	B	1	5B	0	2	1	1	3	0	0	0	0	0	0	3	3	M	1	0	0	0	0	1	0	0	0	0	0	0	0	0	0	0	0	0	0	0	0	0	0	0	0	58
12	F	15	B	1	5B	0	1	1	1	9	0	0	0	0	1	0	9	9	M,T,C	1	0	0	0	0	1	0	0	0	0	0	0	0	0	1	0	1	0	0	0	0	0	0	0	0	56
13	F	37	B	1	5B	0	1	1	1	6	1	1	0	1	1	0	3	3	M,T	1	1	1	0	0	0	0	0	0	0	1	0	1	0	0	0	0	0	0	0	0	0	0	0	0	18
14	F	13	B	1	5B	1	2	1	1	5	0	0	0	0	0	0	6	6	M,T,C	0	1	0	0	0	1	0	1	0	0	0	0	0	1	1	0	0	0	0	1	1	0	0	0	0	17
14	F	13	B	1	5B	0	2	1	1	5	0	0	0	0	0	0	6	3	M	0	0	0	0	0	1	0	0	0	0	0	0	0	0	0	0	0	0	0	0	0	0	0	0	0	56
15	F	12	U	1	5B	1	0	0	0	0	0	0	0	0	0	0	3	3	M	1	0	0	0	0	0	0	0	0	1	0	0	0	0	0	0	0	0	0	0	0	0	0	0	0	41
16	M	23	B	1	5B	1	1	1	1	6	0	0	0	1	1	0	1	1	M	0	0	0	0	0	1	0	0	0	0	0	0	0	0	0	0	0	0	0	0	0	0	0	0	0	47
17	M	23	B	1	5B	0	1	0	0	0	0	0	0	0	0	0	3	3	M	0	0	0	0	0	1	0	0	0	0	0	0	1	0	0	0	0	0	0	0	0	0	0	0	0	38
18	F	13	B	0	5A	0	2	1	1	1	0	0	0	0	0	0	3	3	M,T	0	0	0	0	0	1	0	0	0	0	0	0	0	0	0	0	0	0	0	0	0	0	0	0	0	35
18	F	13	B	1	5B	1	2	1	1	1	0	0	0	0	0	0	3	3	M,T	0	0	0	0	0	1	0	0	0	0	0	0	0	0	0	0	0	0	0	0	0	0	0	0	0	35
19	F	11	B	0	5A	0	2	1	1	3	1	0	0	0	0	0	8	6	M,T,C	1	0	0	0	0	1	0	0	0	0	0	0	0	0	0	0	0	0	0	0	0	0	0	0	0	15
19	F	11	B	0	5A	1	2	1	1	3	1	0	0	0	0	0	8	5	M,T,C	1	0	0	0	0	1	0	0	0	0	0	0	0	0	0	0	0	0	0	0	0	0	0	0	0	21
20	M	14	U	0	5A	1	0	0	0	0	0	0	0	0	0	0	2	2	M,T	1	0	1	0	0	1	0	0	0	0	0	0	0	0	0	0	0	0	0	0	0	0	0	0	0	42
21	F	3	B	0	5A	0	2	2	2	4	0	0	0	1	0	0	1	1	M	1	0	0	0	0	0	0	0	0	0	0	0	0	0	0	0	0	0	0	0	0	0	0	0	0	34
22	M	8	B	1	5B	0	2	1	0	0	0	0	0	0	1	0	8	8	M,T,C	0	0	0	0	0	1	0	0	0	0	0	0	0	0	0	0	0	0	0	0	0	0	0	0	0	42
23	M	7	U	1	5B	1	0	0	0	0	0	0	0	0	0	0	3	3	M	1	0	0	0	0	1	0	0	0	0	0	0	0	0	0	0	0	0	0	0	0	0	0	0	0	43
24	F	6	U	1	5B	0	0	0	0	0	0	0	0	0	0	0	5	5	M,T	1	1	1	0	0	0	0	0	0	0	0	0	0	0	1	0	1	0	0	1	1	0	0	0	0	22
25	F	39	U	1	5B	1	0	0	0	0	0	0	0	0	0	0	4	4	M,T	0	0	0	0	0	0	0	0	0	0	0	0	0	0	0	0	0	0	0	0	0	0	0	0	0	57
26	M	22	B	1	5B	1	1	1	1	3	0	0	0	0	0	0	3	3	M	1	0	0	0	0	0	0	0	0	0	0	0	0	0	0	0	0	0	0	0	0	0	0	0	0	53
27	M	22	B	1	5B	1	1	1	1	3	1	0	0	1	1	0	3	3	M	0	0	0	0	0	0	0	0	0	0	0	0	0	0	0	0	0	0	0	1	1	0	0	0	0	2
28	M	95	B	1	5B	1	1	1	1	8	0	1	1	0	1	0	6	6	M	0	0	0	0	0	0	0	0	0	0	0	0	0	0	1	0	0	0	0	1	1	0	0	0	0	20
29	M	50	U	1	5B	0	0	1	0	0	0	0	0	0	0	1	4	4	M,T,C	1	0	0	0	0	0	0	0	0	0	0	0	0	0	0	0	0	0	0	0	0	0	0	0	0	34
30	F	14	U	0	5A	0	0	0	0	0	0	0	0	0	0	0	2	2	M	0	0	0	0	1	0	1	0	0	0	0	0	0	0	1	0	1	0	0	0	0	0	0	0	0	43
31	F	17	U	1	5B	0	0	1	1	10	0	0	0	1	0	0	2	2	M	0	0	0	0	0	0	0	0	0	0	0	0	0	0	0	0	0	0	0	0	0	0	0	0	0	66
32	M	16	U	1	5B	1	0	0	0	0	0	0	0	0	0	0	7	7	M,T,C	1	1	0	0	0	0	0	0	0	0	0	0	0	0	0	0	0	0	0	0	0	0	0	0	0	32
33	M	9	B	0	4A	0	1	0	0	0	0	0	0	0	0	0	3	3	M,T	1	1	0	0	0	0	0	0	0	0	0	0	0	0	0	0	0	0	0	0	0	0	0	0	0	33
34	F	10	U	1	5B	1	0	0	0	0	0	0	0	0	0	0	3	3	M	0	1	0	0	0	0	0	0	0	0	0	0	0	0	0	0	0	0	0	0	0	0	0	0	0	110
35	F	3	U	0	5A	0	0	0	0	0	0	0	0	0	0	0	3	3	M,T	0	1	1	0	0	0	0	0	0	0	0	0	0	0	0	0	0	0	0	0	0	0	0	0	0	38
36	F	5	U	1	5B	0	0	1	1	4	0	0	0	1	0	0	4	3	M,T	1	0	0	0	0	1	0	0	0	1	0	0	0	0	1	0	0	0	0	1	1	0	0	0	0	5
37	M	5	B	0	5A	0	2	0	0	0	0	0	0	0	0	0	4	4	M,T,C	1	0	0	0	0	1	0	0	0	0	0	0	0	0	0	0	0	0	0	0	0	0	0	0	0	22
38	F	22	B	1	5B	1	1	1	1	4	0	1	0	1	0	0	3	3	M	0	0	0	0	0	0	0	0	0	0	0	0	0	0	0	0	0	0	0	0	0	0	0	0	0	96
39	M	10	U	0	3B	0	0	0	0	0	0	0	0	0	0	0	3	3	C	0	1	0	0	0	0	0	0	0	0	0	0	0	0	0	0	0	0	0	0	0	0	0	0	0	22
40	M	84	U	1	5B	0	0	0	0	0	0	0	0	0	0	0	3	3	M	1	0	0	0	0	0	0	0	0	0	0	0	0	0	1	0	0	0	0	1	1	1	0	0	0	15
41	M	30	B	1	5B	0	1	1	1	3	1	0	0	1	0	0	4	4	M,T	0	0	0	0	0	0	0	0	0	0	0	0	0	0	0	0	0	0	0	0	0	0	0	0	0	22
42	M	31	U	1	5B	1	0	0	0	0	0	0	0	0	0	0	5	5	M,T,C	1	0	0	1	0	1	0	0	0	0	0	0	0	0	0	0	0	0	0	0	0	0	0	0	0	29
43	F	4	B	1	5B	1	1	2	2	3	0	0	0	1	1	0	5	5	M,T,C	0	0	0	0	0	1	0	0	0	0	0	0	0	0	0	0	0	0	0	0	0	0	0	0	0	43
44	F	28	U	1	5B	1	0	1	1	6	0	0	0	0	0	0	6	6	M,T,C	1	1	0	0	0	0	0	0	0	0	0	0	0	0	0	0	0	0	0	0	0	0	0	0	0	21
45	M	4	U	0	5A	0	0	0	0	0	0	0	0	0	0	0	4	4	M,T	0	1	0	0	0	0	0	0	0	0	0	0	0	0	0	0	0	0	0	0	0	0	0	0	0	30
46	M	30	B	1	5A	1	1	1	0	0	0	1	0	0	0	0	3	3	M,C	0	1	0	0	1	0	0	0	0	0	0	0	0	0	1	0	0	0	0	1	1	0	0	0	0	3
47	M	32	B	1	5B	0	2	1	1	6	0	1	0	1	0	0	6	4	M,T	1	1	0	0	0	1	0	0	0	0	0	0	0	0	0	0	0	0	0	0	0	0	0	0	0	51
47	M	32	B	1	5B	1	2	1	1	6	0	1	0	1	0	0	6	5	M,T	1	1	0	0	0	1	0	0	0	0	0	0	0	0	1	0	0	0	0	0	0	0	0	0	0	51
48	M	15	B	1	5B	0	2	0	0	0	0	0	0	1	0	0	3	3	M,T	0	0	0	0	0	0	0	0	0	0	0	0	0	0	0	0	0	0	0	0	0	0	0	0	0	76
48	M	15	B	1	5B	1	2	0	0	0	0	0	0	1	0	0	3	3	M,T	0	0	0	0	0	0	0	0	0	0	0	0	0	0	0	0	0	0	0	0	0	0	0	0	0	76
49	M	6	B	1	5B	0	1	2	2	4	0	0	1	0	0	0	3	3	M	0	0	0	0	0	0	1	0	0	0	0	0	0	0	1	0	0	0	0	1	1	0	0	0	0	23
50	M	21	U	1	5B	1	0	0	0	0	0	0	0	0	0	0	4	4	M,T	1	0	0	0	0	1	1	0	0	0	0	0	0	0	0	0	0	0	0	0	0	0	0	0	0	74
51	M	6	U	1	5B	1	0	0	0	0	0	0	0	0	0	0	2	2	M	0	0	0	0	0	0	0	0	0	0	0	0	0	0	1	0	1	0	0	0	0	0	0	0	0	72
52	F	11	U	1	5B	0	0	1	1	7	0	0	0	1	0	0	5	5	M,T,C	0	0	0	0	0	1	0	0	0	0	0	0	0	0	1	0	1	0	0	1	1	0	0	0	0	9
53	M	18	B	1	5B	1	2	0	0	0	0	0	0	0	0	0	2	1	M	0	0	0	0	0	0	0	0	0	0	0	0	0	0	1	0	1	1	0	0	0	0	0	0	0	71
54	F	6	B	1	5B	1	1	0	0	0	0	0	0	0	0	0	6	4	C,T	1	0	0	0	0	1	0	0	0	0	0	0	0	0	1	0	0	0	0	0	0	0	0	0	0	52
55	M	27	U	0	5A	1	0	0	0	0	0	0	0	0	0	0	2	2	M,T	0	0	0	0	0	0	0	0	0	0	0	1	0	0	1	0	0	0	0	1	1	1	0	0	0	2
56	F	6	B	1	5B	0	1	2	2	4	0	0	0	1	0	0	2	2	M,T	0	0	0	0	0	0	0	0	0	0	0	0	0	0	0	0	0	0	0	0	0	0	0	0	0	33
57	F	7	B	1	5B	0	2	1	1	3	0	0	0	0	0	0	10	5	M,T,C	1	1	0	0	0	0	0	0	0	0	0	0	0	0	0	0	0	0	0	0	0	0	0	0	0	60
57	F	7	B	1	5B	1	2	1	1	3	0	0	0	0	0	0	10	8	M,T,C	1	1	1	0	0	0	0	0	0	0	0	0	0	0	1	0	1	0	0	0	0	0	0	0	0	38
58	F	111	U	1	5B	1	0	0	0	0	0	0	0	0	0	0	3	3	M,T,C	0	0	0	0	0	0	0	0	0	0	0	0	0	0	1	0	1	0	0	0	0	0	0	0	0	5
59	M	16	U	0	2A	0	0	1	0	0	0	0	1	0	0	0	3	3	M,T,C	1	0	0	0	0	0	0	0	0	0	0	0	1	0	0	0	0	0	0	0	0	0	0	0	0	3
60	F	24	B	0	5A	1	1	1	1	4	0	1	0	1	1	0	3	3	M	1	0	0	0	0	0	0	0	0	0	1	0	0	0	0	0	0	0	0	0	0	0	0	0	0	47
61	F	240	B	1	5B	0	2	1	1	2	0	1	0	0	0	0	3	3	M	0	0	0	0	0	0	0	0	0	0	1	0	0	0	0	0	0	0	0	0	0	0	0	0	0	56
62	F	10	U	0	5A	0	0	0	0	0	0	0	0	0	0	0	3	3	M,T,C	0	0	0	0	0	0	0	0	0	0	1	0	0	0	0	0	0	0	0	0	0	0	0	0	0	35
63	M	7	U	1	5B	1	0	0	0	0	0	0	0	0	0	0	3	3	M,T	1	1	0	0	0	0	0	0	0	0	0	0	0	1	0	0	0	0	0	0	0	0	0	0	0	42
64	M	13	U	0	2A	0	0	0	0	0	0	0	0	0	0	0	2	2	M,T,C	0	0	0	0	0	1	0	0	0	0	0	0	0	0	0	0	0	0	0	0	0	0	0	0	0	14
65	F	61	U	1	5B	1	0	0	0	0	0	0	0	0	0	0	7	7	M,T,C	0	1	0	0	0	0	0	0	0	0	0	0	0	0	1	0	0	0	0	1	1	0	0	0	0	15
66	M	25	U	1	5B	0	0	0	0	0	0	0	0	0	0	0	6	6	M,C	0	1	0	0	0	0	1	0	0	0	0	0	0	0	1	0	0	0	1	1	1	1	0	0	0	9
67	M	26	U	1	5B	1	0	0	0	0	0	0	0	0	0	0	5	5	M,T,C	1	1	1	0	0	0	0	0	0	0	0	0	0	0	1	0	1	0	0	1	1	0	0	0	0	19
68	M	39	U	1	5B	1	0	0	0	0	0	0	0	0	0	0	6	6	M,T,C	1	0	1	0	0	0	0	0	0	0	0	0	0	0	1	0	1	0	0	0	0	0	0	0	0	45
69	M	31	B	1	5A	0	1	1	1	3	0	0	0	0	0	0	3	3	M	0	0	0	0	0	0	0	0	0	0	0	0	0	0	0	0	0	0	0	0	0	0	0	0	0	34
70	F	52	B	0	2A	0	1	1	1	.	0	0	0	1	1	0	5	5	M,T,C	0	0	0	0	0	0	0	0	0	0	0	0	0	0	0	0	0	0	0	0	0	0	0	0	0	36
71	M	16	U	1	5B	1	0	1	1	1	0	0	0	0	0	0	3	3	M	1	0	0	0	0	0	0	0	0	0	0	0	0	0	0	0	0	0	0	0	0	0	0	0	0	42
72	F	39	B	0	5B	1	1	1	1	10	0	1	0	0	0	0	3	3	M,T	1	0	0	0	0	0	0	0	0	0	0	0	0	0	1	0	0	0	0	1	1	0	0	0	0	7
73	M	18	B	1	5B	1	1	1	1	7	0	0	0	1	1	0	7	2	M,T	0	0	0	0	0	0	0	0	0	0	0	0	0	0	0	0	0	0	0	0	0	0	0	0	0	46
74	F	25	B	1	5B	0	1	1	1	10	0	0	0	0	0	0	2	2	M,T	1	0	0	0	0	0	0	0	0	0	0	0	0	0	0	0	0	0	0	0	0	0	0	0	0	6
75	F	11	B	1	5B	0	1	0	0	0	0	0	0	0	0	0	4	4	M,T	0	0	0	0	0	0	0	0	0	0	0	0	0	0	0	0	0	0	0	0	0	0	0	0	0	37
76	M	51	U	1	5B	0	0	1	0	0	1	0	1	0	1	0	2	2	M	0	0	0	0	0	0	0	0	0	0	0	0	0	0	0	0	0	0	0	0	0	0	0	0	0	38
77	F	252	U	1	5B	0	0	1	1	.	1	1	0	0	1	0	1	1	M	0	0	0	0	0	0	0	0	0	0	0	0	0	0	0	0	0	0	0	0	0	0	0	0	0	25
78	M	29	B	1	5B	0	1	1	1	2	0	1	0	1	1	0	4	4	M,T	0	0	0	0	0	1	0	0	0	0	0	0	0	0	1	0	0	0	0	1	1	0	0	0	0	6
79	F	59	B	1	5B	0	1	1	1	13	0	0	0	0	0	0	6	6	M,T	0	0	0	1	0	1	0	0	0	0	0	0	0	0	1	1	0	0	0	1	1	0	0	0	0	5
80	M	59	B	1	5B	1	1	1	0	0	0	0	1	1	1	0	6	6	M,T	0	0	0	0	0	0	0	0	0	1	0	0	0	0	1	0	0	0	0	1	1	0	0	0		7
81	F	6	B	1	5B	0	2	0	0	0	0	0	0	0	0	0	3	3	M,T	1	0	0	0	0	0	0	0	0	0	0	0	0	0	0	0	0	0	0	0	0	0	1	0	Pineal	30
81	F	6	B	1	5B	1	2	0	0	0	0	0	0	0	0	0	3	3	M,T	1	0	0	0	0	0	0	0	0	0	0	0	0	0	0	0	0	0	0	0	0	0	1	0	Pineal	30
82	M	14	U	1	5B	1	0	0	0	0	0	0	0	0	0	0	4	4	M,T	0	0	0	1	0	0	0	0	0	0	0	0	0	0	0	0	0	0	0	0	0	0	0	0	0	43
83	F	8	B	0	5A	1	2	0	0	0	0	0	0	0	0	0	2	2	M,T,C	0	0	0	1	0	0	0	0	0	0	0	0	0	0	0	0	0	0	0	0	0	0	0	0	0	36
84	M	32	U	1	5B	0	0	0	0	0	0	0	0	0	0	0	6	6	M,T	0	0	0	0	0	0	0	0	0	0	0	0	0	0	1	0	0	0	0	1	1	0	0	0	0	12
85	F	47	U	1	5B	0	0	1	1	3	0	0	0	1	0	0	3	3	M,T	1	0	0	0	0	0	0	0	0	0	0	0	0	0	0	0	0	0	0	0	0	0	0	0	0	37
86	F	41	B	1	5B	0	1	1	1	1	1	1	0	0	1	0	3	3	M,T	0	0	0	0	0	0	0	0	0	0	0	0	0	0	1	1	0	0	0	1	1	0	0	0	0	3
87	M	26	U	1	5B	0	0	1	1	4	0	0	0	0	0	0	3	3	M,T,C	1	0	0	0	0	0	0	0	0	0	0	0	0	0	0	0	0	0	0	0	0	0	0	0	0	19
88	M	18	B	1	5B	0	2	1	1	2	1	0	0	0	0	0	3	3	M,T	0	1	0	0	0	1	0	0	0	0	0	0	0	0	1	0	0	1	1	1	1	0	0	0	0	5
89	M	4	B	1	5B	1	2	2	2	2	0	0	0	0	0	0	6	3	M,T,C	1	0	0	0	0	1	0	0	0	0	0	0	0	0	0	0	0	0	0	0	0	0	0	0	0	18
90	F	8	B	0	4A	0	2	1	1	4	0	0	0	0	0	0	3	2	M	1	0	0	0	0	0	0	0	0	0	0	0	0	0	0	0	0	0	0	0	0	0	0	0	0	30
90	F	8	B	0	3B	1	2	1	1	4	0	0	0	0	0	0	3	3	M,T	1	0	0	0	0	0	0	0	0	0	0	0	0	0	0	0	0	0	0	0	0	0	0	0	0	30
91	F	6	B	1	5B	0	2	0	0	0	0	0	0	0	0	0	6	3	M	0	0	0	0	0	1	0	0	0	0	0	0	0	0	0	0	0	0	0	0	0	0	0	0	0	54
92	F	50	U	1	5B	0	0	0	0	0	0	0	0	0	0	0	9	9	M,T,C	1	0	0	1	0	0	0	0	0	0	0	0	0	0	1	1	1	0	0	0	0	0	0	0	0	56
93	M	120	B	1	5B	0	1	1	1	.	0	0	0	0	0	0	3	3	M	0	1	0	0	0	0	0	0	0	0	0	0	0	0	1	0	0	0	0	1	1	0	0	0	0	30
94	M	13	B	1	5B	1	1	1	1	6	0	0	0	0	0	0	8	8	M,T,C	1	1	0	0	0	0	0	0	0	0	0	0	0	0	1	0	0	0	0	1	1	0	0	0	0	42
95	F	57	U	1	5B	1	0	1	1	4	0	0	0	0	0	0	7	7	M,T,C	0	0	0	0	0	0	0	0	0	0	0	0	0	0	1	0	1	0	0	0	0	0	0	0	0	64
96	F	4	B	0	5A	1	1	0	0	0	0	0	0	0	0	0	5	5	M,T,C	1	0	0	0	0	0	0	0	0	1	0	0	0	0	0	0	0	0	0	0	0	0	0	0	0	35
97	F	6	B	1	5B	1	1	0	0	0	0	0	0	0	0	0	3	3	M	0	1	1	0	0	0	0	0	0	0	0	0	0	0	0	0	0	0	0	0	0	0	0	0	0	56
98	F	8	B	0	5A	0	2	0	0	0	0	0	0	0	0	0	3	3	M,T,C	0	0	0	0	0	0	0	0	0	0	0	0	0	0	0	0	0	0	0	0	0	0	0	0	0	25
99	F	11	B	1	5B	1	1	1	0	0	0	0	0	0	0	1	3	3	M,T,C	0	0	0	0	0	1	0	0	0	0	0	0	0	0	0	0	0	0	0	0	0	0	0	0	0	16
100	M	4	B	1	5B	1	2	2	2	2	0	0	0	3	3	0	5	3	M,T	0	0	0	0	0	0	0	0	0	0	0	0	0	0	0	0	0	0	0	0	0	0	0	0	0	26
101	F	5	B	1	5B	1	2	0	0	0	0	0	0	0	0	0	7	4	M,T,C	0	0	0	0	0	0	0	0	1	0	0	0	0	0	0	0	0	0	0	0	0	0	0	0	0	27
102	M	12	B	0	3A	1	2	1	1	6	0	0	0	1	0	0	6	3	M,T,C	0	0	0	0	0	1	0	0	0	0	0	0	0	0	0	0	0	0	0	0	0	0	0	0	0	23
102	M	12	B	0	3A	0	2	1	1	6	0	0	0	1	0	0	6	3	M,T,C	1	0	0	0	0	1	0	0	0	0	0	0	0	0	0	0	0	0	0	0	0	0	0	0	0	23
103	M	9	B	1	5B	1	2	0	0	0	0	0	0	0	0	0	8	3	M,T,C	0	0	0	0	0	1	0	0	0	0	0	0	0	1	0	0	0	0	0	0	0	0	0	0	0	27

S1 Supplementary Table. Complete de-identified data set used for analyses.
